# French Jurisprudence

**Published:** 1867-01

**Authors:** 


*French Jurisprudence.*—Duke Caderousse-Gramont died a short time ago of phthisis, leaving the bulk of his large fortune to his physician, Dr. Déclat. The Duke's prodigality had been so notorious that the family had interfered a few years ago and obtained some control over the property. Piqued at these proceedings, the deceased had intended, by his will, to frustrate the rightful heirs. The latter attacked the will, and easily had it declared null and void, as by the French law, the medical man in attendance on a testator cannot be made heir to the latter.—*Ibid.*

